# Identification of tissue of origin in carcinoma of unknown primary with a microarray-based gene expression test

**DOI:** 10.1186/1746-1596-5-3

**Published:** 2010-01-13

**Authors:** Federico A Monzon, Fabiola Medeiros, Maureen Lyons-Weiler, W David Henner

**Affiliations:** 1 Clinical Genomics Facility, University of Pittsburgh, Pittsburgh, Pennsylvania, USA; 2 Department of Laboratory Medicine and Pathology, Mayo Clinic, Rochester, Minnesota, USA; 3 Pathwork Diagnostics, Sunnyvale, California, USA

## Abstract

**Background:**

Carcinomas of unknown primary (CUP) represent approximately 3%-5% of malignant neoplasms. Identifying the tissue of origin (TOO) in these tumors allows for more specific treatment and improves outcomes. However, primary classification remains a challenge in many cases. We evaluated the ability of a microarray-based gene expression test to identify the TOO in tumor specimens from 21 patients with a diagnosis of CUP.

**Methods:**

The Pathwork^® ^TOO Test was used to measure gene expression patterns for 1550 genes; these were compared for similarity to patterns from 15 known tissue types.

**Results:**

The TOO Test yielded a clear single positive call for the primary site in 16 of 21 (76%) specimens and was indeterminate in 5 (24%). The positive results were consistent with clinicopathologic suggestions in 10 of the 16 cases (62%). In the remaining six cases the positive results were considered plausible based on clinical information. Positive calls included colorectal (5), breast (4), ovarian (3), lung (2), and pancreas (2). The TOO Test ruled out an average of 11 primary tissues in each CUP specimen.

**Conclusion:**

The Pathwork TOO Test reduced diagnostic uncertainty in all CUP cases and could be a valuable addition or alternative to current diagnostic methods for classifying uncertain primary cancers.

## Background

Patients with carcinoma of unknown primary (CUP) present with metastatic disease for which the tissue of origin (TOO) cannot be identified. About 3%-5% of all diagnosed cancers are classified as CUP [1-4] and an estimated 31,490 new cases of cancer of unspecified primary sites were diagnosed in the United States in 2008 [5]. Prognosis of patients with CUP is usually poor with empiric treatment. Median survival is 3-9 months even with newer combination regimens [4,6-10]. It has been shown that survival can improve if the primary site is identified and specific therapy is instituted [11,12] as currently recommended in therapeutic guidelines [4,13].

Unfortunately, primary tumor detection remains challenging. While serum tumor markers, imaging tests, and immunohistochemistry (IHC) panels can help identify the tissue of origin, the primary site is identified in fewer than 30% of those who present initially with occult primary tumor [13-15]. Furthermore, some positive findings can be misleading [2,16]. For example, in three large IHC studies (>50 specimens) of known metastatic specimens, IHC findings failed to agree with the site of origin in about one third of cases [17-19]. In addition, CUP diagnostic workups today are all too often time-consuming, expensive, and unsuccessful [13,20].

Recently, gene expression tests to classify tumors by tissue origin have been developed. These tests employ microarrays or real-time reverse transcriptase polymerase chain reaction (RT-PCR) to measure mRNA transcripts [21-29] and one uses a microarray to quantify microRNAs [30]. Thus far, performance of these expression tests has been assessed mainly by challenges against panels of tumors from known primary sites; however, the panel composition has varied widely in terms of specimen number, specimen handling, tissue types included, number of replicates for each tissue type, and the proportions of metastatic and poorly differentiated tumors. Overall, the accuracy in these studies has been in the range of 76% to 89%. Although these performance studies are necessary and important, a challenge with actual CUP specimens is needed to gauge the true clinical value of these tests. Designing such a study is inherently difficult because CUP specimens, by definition, lack a reference diagnosis. Only recently have expression-based tissue test results in CUP specimens been reported [31-33].

The Pathwork^® ^TOO Test (Pathwork Diagnostics, Sunnyvale, California, U.S.) is a microarray-based gene expression diagnostic test for determining the similarity of a tumor specimen to 15 known tissue types. The test interprets the expression of 1550 genes in each specimen by applying normalization and classification algorithms to gene expression data from a microarray. The similarity of each tumor specimen's gene expression pattern is compared to the 15 tissues covered by the test. For each specimen, the pathologist receives a report with 15 separate scores that reflect the similarity of the specimen's gene expression profile to each of the reference tissues. Evaluation of the TOO Test found highly reproducible results across four laboratories [28] and in a blinded multicenter evaluation of 547 known primaries (47% metastatic, 53% poorly differentiated or undifferentiated primaries), the test had an overall agreement of 87.8% with the pathologist-issued diagnosis [29].

In this study, we evaluated the clinical utility of the TOO Test in identifying the primary site in specimens from patients diagnosed with CUP. The aims were to evaluate the test's ability to issue a clear positive call in classic CUP specimens, to check the consistency of the test results against a short-list of diagnostic possibilities based on clinicopathology, and to estimate the potential added clinical value of positive and negative results in guiding management.

## Methods

### Study Design

This was a retrospective study of tumor specimens from 21 patients diagnosed with CUP. Fresh-frozen specimens were obtained from tissue banks at the Mayo Clinic and the University of Pittsburgh. The specimens had been archived between January 1998 and December 2006. Specimen processing and microarray scanning were performed at each site. Data files were analyzed by Pathwork Diagnostics and a report was generated. Electronic test scores were sent to study site pathologists who were blinded to the associated patient's clinicopathologic information. The pathologists used predetermined cutoffs to make final expression test calls of positive or indeterminate for each specimen. The code was then broken and expression results were compared with the available clinical, radiologic, pathological, and therapeutic findings as a basis for conclusions about the consistency, plausibility, and potential added clinical value of TOO Test results. The study was conducted under protocols approved by the institutional review boards of each institution.

### Case Selection and CUP Definition

Cases were first identified for possible inclusion in the study by manual and electronic searching of tissue bank archives. Search terms and diagnostic codes included "CUP," "unknown," and "uncertain." Specimens with a high number of IHC results were also selected for review.

Most patients whose specimens were included had received a complete history, physical, and full clinical, laboratory, imaging, and pathologic workups, including standard histologic and IHC examination, prior to their designation as CUP. Specimens from patients from the Mayo Clinic (n = 11) were also evaluated by a panel of stains to rule out certain subsets of malignancies. These stains included but were not limited to: keratins or epithelial membrane antigen, S-100 or HMB45, LCA (CD45), chromogranin or synaptophysin, CK20, CK7, thyroid transcription factor 1 (TTF-1), and CDX-2. Directed evaluation of symptomatic areas and targeted testing based on history or symptoms (e.g., abdominal CT, mammogram in women, colonoscopy in cases of liver metastasis) were undertaken in Mayo Clinic patients. Specimens designated as biopsy-proven carcinomas (e.g., carcinomas, adenocarcinomas, and mucinous adenocarcinomas) with varying levels of differentiation and from various sites were included. Available treatment data and performance status for Mayo Clinic patients were compiled but not required for inclusion.

The minimum inclusion criteria for screened specimens were: (1) characterization as CUP after well-documented pathologic examination; (2) availability of frozen specimen block with histologic verification of at least 60% tumor representation and less than 20% necrosis; (3) availability of medical records with core demographics as well as a clinicopathologic report including a summary of any narrowed diagnostic possibilities. Any specimens assigned a definitive single tissue type or included in previous studies reporting performance of the TOO Test [28,29] were excluded.

### Gene Expression Test

**Figure 1:**
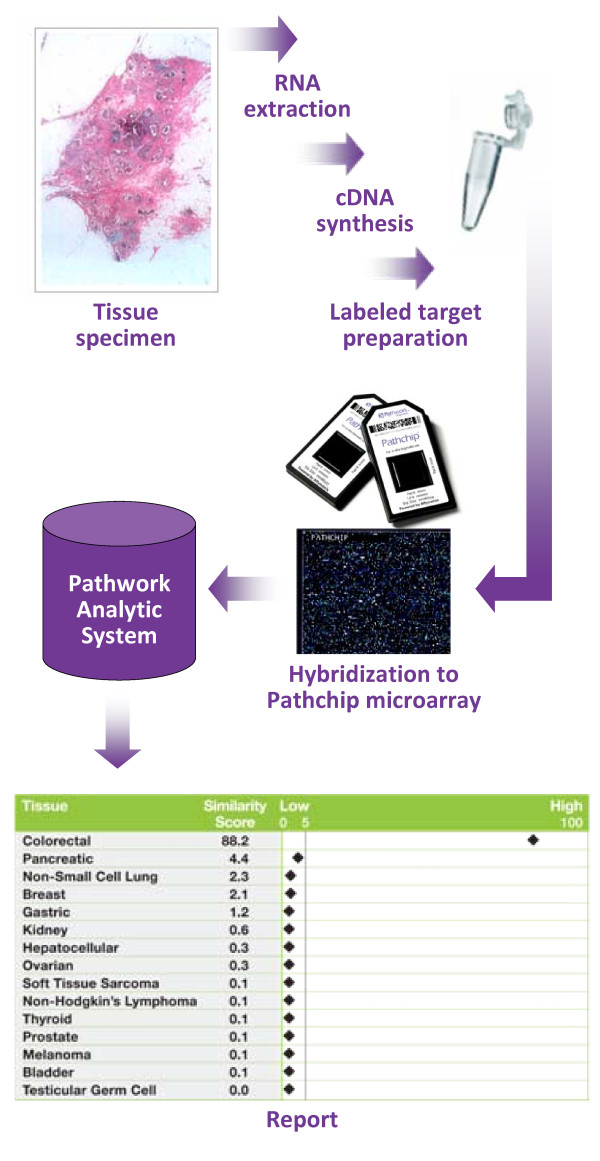
**Tissue of Origin Test workflow and results**. RNA from frozen tissue is extracted, amplified, and biotin-labeled before hybridization to a Pathchip™ microarray. Gene expression data are then analyzed and a report indicating the studied tissue's molecular similarity to established profiles for 15 tissues of origin is produced.

The study pathologist at each site checked necrosis and tissue viability in a section from each frozen tissue block and then RNA was extracted and processed according to previously published methods [28]. Spectrophotometry was used to assess adequate total RNA concentration and purity. In general, the protocol for processing the RNA, amplifying and labeling fragments, hybridizing material on the microarray, and scanning is similar to the standard Affymetrix protocol for 3'-based gene expression analysis. Both laboratories used the proprietary Pathwork Pathchip™ or an Affymetrix GeneChip array on Affymetrix 3000 or 3000Dx GeneChip instrumentation (fluidics station and scanner) and the GeneChip operating software to generate gene expression data (.CEL files). Raw data files were submitted to Pathwork Diagnostics for automated analysis and report generation (Figure 1).

The Pathwork TOO Test algorithm transforms probe-level intensity data into gene expression values, performs data verification, and standardizes expression values using a 121-gene standardization method whose performance has been previously described [28,34]. Expression levels of the 1550 genes for each specimen are then compared in pairwise fashion with the pre-established gene profiles for each of the 15 tissues on the test panel. The results are presented on an electronic report as 15 separate Similarity Scores (SS), one for each tissue on the panel.

### Data Analysis

Coded electronic reports with SS and no patient information were sent to investigators, who used pre-established cutoffs to make a final determination about each tissue type as positive or indeterminate. The SS for each of the 15 tissue types ranged from 0 (very low similarity) to 100 (very high similarity). Per cut-offs determined before the study, a SS of 30 or above indicated the presence of a given tissue in the specimen, a SS of 5 or less indicated the absence of a given tissue in the specimen, and a SS between 5 and 30 was considered indeterminate [28,29].

For each specimen, the final TOO Test result was compared with available clinical, laboratory, and imaging results for that specimen and a determination was made about the overall consistency of the TOO Test positive result with the clinicopathologic suggestions. Given the challenging nature of CUPs, inconsistencies were expected. In some inconsistent cases, investigators evaluated the plausibility of TOO Test results by re-examining the patient file and, in some cases, performing additional IHC analysis (CDX2 stain in two cases). Investigators also performed a case-by-case review to determine the consistency of negative tissue calls with the clinicopathologic suggestion.

## Results

### Patient and Tumor Characteristics

Characteristics for individual patients from Mayo (n = 11) and the University of Pittsburgh (n = 10) are detailed in Table 1. Most patients were in the age range of 60-69 (n = 6) or 70-79 years of age (n = 7). The majority of patients (15 of 21) were female. Tumor specimens were taken from over a dozen different biopsy sites. Most cases were described as having a stage IV or metastatic tumor. Most specimens were characterized morphologically as poorly differentiated (n = 13) or moderately differentiated (n = 7).

**Table 1:** Patient characteristics

**Case No**.	Age	Gender	Biopsy site	Prior cancer history	Morphology	CUP treatment regimen
1	70-79	F	Liver	Breast	Moderately-differentiated adenocarcinoma	supportive care only

2	70-79	M	Mesentery	None	Poorly-differentiated adenocarcinoma	supportive care only

3	60-69	F	Soft Tissue	Breast	Poorly-differentiated carcinoma	supportive care only

4	50-59	F	Peritoneum	None	Poorly-differentiated adenocarcinoma	paclitaxel/carboplatin/gemcitabine

5	80-89	F	Bone	Breast	Poorly-differentiated carcinoma	radiation

6	80-89	F	Soft tissue	None	Moderately-differentiated carcinoma	supportive care only

7	80-89	M	Pleura	Prostate, Bladder	Moderately-differentiated adenocarcinoma	supportive care only

8	60-69	F	Peritoneum	None	Poorly-differentiated carcinoma	paclitaxel/carboplatin

9	70-79	F	Peritoneum	Rectal	Poorly-differentiated carcinoma	supportive care only

10	50-59	F	Peritoneum	Ovarian	Well-differentiated mucinous adenocarcinoma	supportive care only

11	60-69	F	Peritoneum	Breast	Poorly-differentiated carcinoma	paclitaxel/carboplatin

12	50-59	F	Right Femur	N/A	Metastatic, well-to- moderately differentiated adenocarcinoma	N/A

13	50-59	F	Left Femur	N/A	Metastatic, poorly-differentiated adenocarcinoma	N/A

14	70-79	F	Lymph Node	N/A	Metastatic poorly-differentiated adenocarcinoma	N/A

15	70-79	F	Lung (Upper Lobe)	N/A	Moderately-differentiated papillary adenocarcinoma	N/A

16	60-69	F	Omentum	N/A	Metastatic poorly-differentiated adenocarcinoma	N/A

17	70-79	M	Abdominal Wall	N/A	Metastatic poorly-differentiated adenocarcinoma	N/A

18	70-79	M	Right Groin	N/A	Metastatic adenocarcinoma	N/A

19	40-49	M	Colon/Omentum	N/A	Poorly-differentiated adenocarcinoma	N/A

20	60-69	M	Right Clavicle	N/A	Moderately-differentiated mucinous adenocarcinoma	N/A

21	60-69	F	Liver/Lymph Node	N/A	Liver: poorly-differentiated carcinoma LN: metastatic poorly-differentiated carcinoma	N/A

**N/A **= not available

### Tumor IHC Analysis

A total of 49 unique IHC markers were employed in an attempt to identify the origin of the tissue in these 21 specimens. An average of six IHC tests were performed on each specimen (range: 0 to 13) and 12 of the 21 specimens received at least seven different IHC staining tests including those targeting proteins known to be useful in predicting tissue type (e.g., CK7, CK20, ER, PSA, TTF-1) [18,19]. Results of IHC analyses are listed in Table 2.

**Table 2:** Immunohistochemical and Tissue of Origin Test results

	Immunohistochemistry Results			Tissue of Origin (TOO) Test results				
			
**Case No**.	Negative	Positive	Suspected tissues based on clinicopathology	TOO result	TOO SS	Rule out tissues	Consistent	Management Value
1	BRST-2, CK20, TTF-1, ER, PR, Her2/neu	CK7	Pancreas, Breast, Upper GI	Indeterminate	22.1	8	--	--
2	PSA	CK20, CK7, CDX2*	Kidney	Colorectal	83.7	14	No*	Yes
3	BRST-2, CK20, TTF-1, ER, PR, Her2/neu	CK20, CK7, WT1	Ovary or Breast	Ovary	87.6	14	Yes	Yes
4	chromogranin, synaptophysin, calretinin, TTF-1, PR	CK20, CK7, CDX-2, CK5/6, MOC31, ER, CEA	GI Primary, Colorectal	Colorectal	81.8	13	Yes	Yes
5	CDX-2, CD31, CK20, Melan A, S-100, TTF-1	AE1/AE3, CK7	Renal cell carcinoma	Indeterminate	24.7	11	--	--
6	Thyroglobulin	TTF-1	Lung, Thyroid	Breast	61.2	12	No*	Yes
7	CK20, PSA, TTF-1	CK7, AE1/AE3, keratin 903, p63	Lung	Indeterminate	23.6	8	--	
8	BRST-2, CD10, CD45, C-kit	CDX-2, CAM5.2, CK20, CK7, Keratin	Colorectal	Colorectal	64.1	12	Yes	Yes
9	CK20, CK7, TTF-1, ER, PR		Colorectal, Ovary	Lung	35.5	10	No*	Yes
10		CDX-2*, CK20, CK7	Colorectal, Ovary	Colorectal	71.2	13	Yes	Yes
11	ER, PR, Her2/neu, BRST-2, CDX-2, CK20	CK7, p53, WT1	Breast, Ovary	Ovarian	86.6	14	Yes	Yes
12	chromogranin, synaptophysin, surfactant, TTF, CK20, LCA, CA-125, Her2/neu	ER, PR, monoclonal CEA, focal CK7	Breast, Ovary, Uterine	Breast	96.4	14	Yes	Yes
13	Her2/neu, PR	estrogen receptor (10% cells staining at 1+)	Breast	Breast	95	14	Yes	Yes
14	HMB45, Tyrosinase, Melan-A, RCC, ER	CK7, AE1/AE3, CAM 5.2, TTF, S100, surfactant protein, CK20; weak CDX-2	Lung	Ovarian	87.5	13	No*	Yes
15		EGF-R	Lung	Lung	66.9	12	Yes	Yes
16	LeuM1, S100	PLAP, CA-125, monoclonal CEA	Pancreas, Ovary	Pancreas	39.5	11	Yes	Yes
17	PSA, neuroendocrine markers, S100	mucicarmine, CEA, cytokeratin	Colon, Prostate, Stomach, Lung	Pancreas	37.3	10	No*	Yes
18	PSA, TTF, calretinin, thrombomodulin, P63	CK7, CK20, monoclonal CEA, CDX-2	Esophagus, Stomach, Pancreas, Biliary	Breast	36.1	10	No*	Yes
19	smooth myo actin, desmin, S100, HMB-45, CD34, chromogranin, CK7	focal C-kit, focal synaptophysin, focal CK20, mucicarmine, PAS	Colon	Colorectal	31.5	8	Yes	Yes
20	Not available	Not available	None	Indeterminate	24	9	--	--
21	mucicarmine, HepPar, alpha-fetoprotein, synaptophysin	CEA monoclonal/polyclonal, Cam-5.2, AE1	Liver, Cholangiocarcinoma	Indeterminate	20.7	9	--	--

* Still a plausible primary call given the overall patient presentation and clinicopathologic findings; see text for discussion.

SS: Similarity Score; AE: cytokeratin; BRST: Breast; C-kit: cytokine receptor CD117; CA-125: cancer antigen 125; Cam 5.2: anti-cytokeratin; CD: cluster of differentiation; CDX: an intestine-specific transcription factor; CEA: carcinoembryonic antigen; CK: cytokeratin; ER: estrogen receptor; HepPar: hepatocyte paraffin; Her-2/neu: human epidermal growth factor receptor 2; HMB-45: human melanoma black; LeuM1: a granulocyte-related differentiation antigen; P53: tumor protein 53, a transcription factor; P63: a P53-related nuclear protein; PAS: periodic acid Schiff stain; PLAP: placenta-like alkaline phosphatase; PR: progesterone receptor; PSA: prostate-specific antigen; RCC: renal cell carcinoma marker; S-100: a neuroectodermal antigen; TTF-1: thyroid transcription factor-1; WT1: Wilms' tumor protein-1

### TOO Test Results

**Figure 2:**
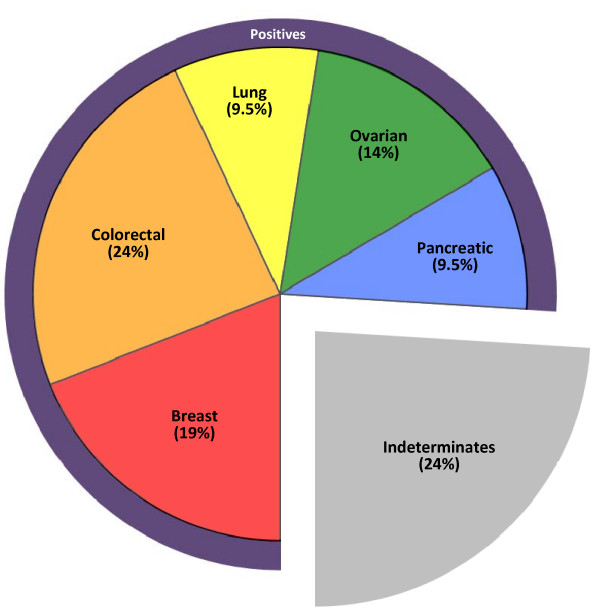
**Distribution of tumor types in 21 CUPs**. The Tissue of Origin Test called the majority of specimens definitively positive for a single tumor type; in the indeterminate calls, the TOO Test ruled out an average of nine tissue types per specimen.

The Pathwork TOO Test gave a positive result for a single tissue in 16 (76%) of 21 CUP specimens (Table 2). The identified primary sites included colorectal (5), breast (4), ovary (3), lung (2), and pancreas (2). The TOO Test was indeterminate in five cases (24%) (Figure 2). The average SS in positive calls was 66.4 while the average highest SS in indeterminate calls was 23.0.

The TOO Test positive results were consistent with clinicopathologic suggestions in 10 of the 16 cases (62%). For example, in Case 3 the TOO Test yielded a positive ovarian call, thus confirming one of the two main tissues types under consideration (breast vs. ovarian). In Case 4, the TOO Test pointed to a colorectal origin while the pre-existing clinicopathologic data (based on colonoscopy, mammogram, abdominal CT, and 10 IHC stains) hinted at, but did not confirm, a gastrointestinal (GI) origin. In five of seven specimens called positive for GI origin (5 colorectal, 2 pancreas), the TOO Test result was not unexpected given the pre-existing clinical differential.

Overall, six cases of unexpected positive TOO Test results were produced. There were no obvious instances of TOO Test error or high unlikelihood based on all existing patient information (e.g., a prostate call in a female patient, a sarcoma or melanoma call for a histologically confirmed adenocarcinoma). Also, in many cases, tissue types under active consideration were "ruled out" based on negative results in expression testing. In Case 6, for example, where the patient was strongly suspected of having either a primary lung or thyroid carcinoma due to positive TTF-1 staining, the TOO Test result of breast carcinoma, while unexpected, had not been absolutely ruled out by clinical factors. Importantly, TTF-1 positivity has been reported in a small percentage of breast and colon carcinomas [35,36]. Further, the TOO Test gave negative Similarity Scores for both lung (SS = 3.8) and thyroid (SS = 1.1). Similarly, in Case 2 a diagnosis of renal cell carcinoma was originally suspected but the TOO Test call of colorectal carcinoma was not improbable and the TOO Test also provided a negative kidney signal (SS = 0.6). Immunostain for CDX2 performed during this study and not available at the time of diagnosis was positive, supporting a colorectal origin. On average, the TOO Test ruled out 11 tissue types per case, including in the indeterminate cases. In cases with a positive call, the average number of tissue types ruled out (i.e., SS <5) was 12 while the average in indeterminate calls was 9.

## Discussion

For patients presenting with uncertain primary tumors and who do not have a primary site identified even after exhaustive investigation empiric chemotherapy is rarely successful and often quite intolerable [7-9]. Better survival is achieved when tissue-specific treatment strategies are utilized [11,12,33]. Thus, availability of a test that identifies the tissue of origin would increase the chances of a patient receiving a more targeted and less toxic therapy. Depending on how such a test is integrated into the workup of uncertain primaries, it may also reduce the overall time and expense associated with the hunt for a primary tumor. In addition, as new tissue-targeted therapies are expected to be introduced, correct identification of the primary site will become even more important in guiding optimal patient management.

Recently, we reported that the Pathwork Tissue of Origin test showed robust performance in a large validation study with 547 tumors of known origin [29]. We now have evaluated the performance of this test in a cohort of 21 CUP cases. In this cohort the Pathwork TOO Test was able to identify a probable single primary site in 16 (76%) of the cases. These results suggest that the TOO Test can significantly reduce diagnostic uncertainty in patients with CUP.

Three recent studies have reported on gene expression profiling as a strategy to determine TOO in patients with CUP [31-33]. Two of these studies employed a microarray-based 495-gene-expression classifier (CupPrint, Agendia, Amsterdam, NED), which gave a confident and clinically valuable result in 14 (64%) of 22 CUPs from one study [32] and a clinically feasible result in 18 (86%) of 21 CUPs from the other study [31]. The third study evaluated a 10-gene RT-PCR-based expression assay (Veridex, La Jolla, CA USA) and identified a TOO in 23 (62%) of the 37 specimens classified as CUP after IHC analysis [33]. Thus, expression tests with widely varying designs and features (e.g., number/types of genes; algorithm strategies; training set size; specimen handling protocols; number of tissue types on panel) have recently demonstrated an ability to issue a classification for a majority of CUPs.

This general uniformity in call rates for identifying a TOO in CUP patients among the various expression tests (i.e., 62%-86%) does not necessarily imply a uniformity of call accuracy or assay range. By definition, the gold standard for tumor calls in CUP cases is unknowable. Thus, accuracy in tissue identification can only be evaluated with the use of a large and diverse set of known tumor specimens. This type of evaluation is needed before any projections can be made about a test's clinical diagnostic value--much less its potential impact on therapy choices and outcome--in the setting of CUP. For example, in one of the studies cited above [32], a parallel analysis of 84 tumors with known origin revealed total assay accuracy of 83%; however, the test misclassified 7 of 11 lung tumors and 3 of 3 pancreas tumors. Thus, if we extrapolate these results to CUP specimens, a large number of CUP tumors with lung or pancreas origin could yield a positive, albeit incorrect, tissue identification. Due to the small number of studied samples (in particular of the lung and pancreas groups) it is not possible to determine the performance of that test for individual tissue types. This same assay showed an accuracy of 87% in a previous study with 119 tumor samples; however, some tissue types were represented by only one or two specimens (e.g., breast, adrenal) [27]. The 10-gene RT-PCR assay showed an accuracy of 76% in a set of 48 metastatic samples [26]. Importantly, in a less rigidly defined set of 120 CUP specimens analyzed with the RT-PCR assay [33], this test could not yield a result (due to insufficient mRNA quality or yield) or failed to assign a TOO (perhaps due to a limited 6-tissue panel) in 48% of the cases. When the Pathwork TOO Test was validated in a large multicenter study (n = 547), of poorly differentiated and undifferentiated primary cancers and metastatic tumors, it showed an overall sensitivity (positive percent agreement with reference diagnosis) of 87.8% for the 15 tissues of origin included in the panel [29]. In this validation study, each of the 15 different tissue sites was represented by at least 25 specimens. These performance characteristics in known tissues must be considered when assessing the likelihood of expression test accuracy in CUP cases.

Our study has several limitations, chief among them the lack of a gold standard for comparison. This inability to confirm accuracy, which is unavoidable in CUP diagnostic studies, only heightens the importance of quality and rigor in the associated clinical and pathological investigations. In this study, patients and CUP specimens were characterized in clinics with extensive experience in oncology workups. Full histories, imaging results, lab records, and pathology reports (including an average of six IHC performed in each case) were available before the CUP diagnosis was issued. Thus, within the inherent constraints of CUP study design, this study provides a fair measure of test performance--certainly at least as fair as any of the seminal validation studies of IHC itself [17-19]. Another limitation of this study was its small size. This is related to the difficulty in finding well-characterized fresh-frozen CUP specimens. Using frozen tissue as a basis for initial CUP performance testing makes sense since it yields more intact mRNA [37]; however, we recognize that it is necessary to validate the TOO Test with formalin-fixed paraffin embedded (FFPE) specimens in order to allow the test to be applied widely in non-research clinical settings.

Given the absence of a gold standard to evaluate CUP assay performance, a surrogate marker of accuracy utilized by prior studies has been the correlation of the TOO prediction with existing clinicopathologic information [31-33,38]. In our study, 10 cases show consistency with the clinicopathologic-based differential diagnosis (Table 2) and in six cases the test's suggested diagnosis was inconsistent. Review of cases where TOO Test positive results were not consistent with IHC and/or tentative clinical suggestions sheds further light on the trustworthiness of gene expression profiles. None of these six TOO Test results were deemed implausible or in absolute contradiction to any known clinicopathologic findings. Even the TOO Test call of breast cancer in a male (Case 18), while surprising, cannot be considered patently incorrect given current estimates of approximately 2000 such new cases in the U.S. every year with a rising incidence [39] and also considering reports of male breast cancer presenting as CUP [40]. In addition, the accompanying TOO Test negative results add weight to the plausibility of surprising positive calls by ruling out several of the originally suspected primaries. In fact, the availability of "rule outs" is a unique feature of TOO Test design that may eventually prove valuable in case management; other tests provide the result of the most molecularly similar tumor but do not report information that allows ruling out specific tissue types. Thus, although six of the TOO Test results were unexpected, given the published performance characteristics of the test and recalling the performance deficits of IHC as cited previously, they were still clinically plausible TOO sites. Based on the overall clinical contributions of TOO Test results, we estimated that the expression test would have helped to inform patient management decisions in the majority of cases (Table 2): definitively in the 16 positive cases by identifying a single primary site, and to varying degrees in the five indeterminate cases by eliminating potential primary sites.

Another measure of the clinical relevance of TOO identification is the ability to evaluate response to therapy in those tumors that were treated with tissue-specific approaches. In this regard, Varadhachary and co-workers reported that patients with a molecular signature of colon carcinoma showed better response to colorectal-specific therapies than to empiric CUP therapy [33]. Unfortunately, the size of our study and the unavailability of full outcome and treatment information for all 21 patients make it impossible for us to quantify how the expression results might have translated into changed therapy or improved outcomes. However, review of the treatment data available for about half of the cases indicated that most patients got the type of supportive care with or without empiric chemotherapy that is typical in CUP [4,7-10]. Of the 11 Mayo patients, for example, four received at least 4 cycles of chemotherapy and one received radiation therapy (4 radiation sequences, 800 cGy). Many of these patients likely would have received a more tissue-specific therapy if a trusted expression test result had been available at presentation. In Case 11, for example, one of the options for more ovarian-focused chemotherapy might have been given instead of paclitaxel/carboplatin if the TOO Test result of ovarian cancer had been available. In Case 6, doxorubicin and paclitaxel might have been given rather than only supportive care if the TOO Test result supporting a breast origin had been known. Similarly, in Cases 4 and 8, patients might have received bevacizumab instead of a broad-based chemotherapy (paclitaxel/carboplatin with or without gemcitabine) if the TOO Test result indicating colorectal cancer had been available at the time of diagnosis.

Recently, based on retrospective and prospective analyses [12,33], some researchers have already advocated using expression profiles to guide targeted therapy in patients with colorectal cancer profiles. Such changes in CUP therapy directed by molecular profiling might reasonably be expected to equate to improvement in outcomes, but this important hypothesis needs to be tested in prospective studies. Only carefully designed studies will reveal whether tumors originally deemed CUPs respond to specific treatment in the same manner as more well-differentiated tumors of the same tissue type. It may be, for example, that hard-to-identify CUP-like tumors actually possess distinct genetic/phenotypic aberrations (while maintaining tissue-specific expression traits) that limit their susceptibility to therapies tested only in non-CUP tumors [41,42]. In this context, because of the TOO Test's high accuracy in poorly differentiated tumor types and also because of the high number of genes assayed, this test may be particularly well suited not only to classifying tissue origin in CUP but, in the future, to providing information about the tumor's susceptibility to specific therapies.

## Conclusions

The Pathwork TOO Test shows clear promise in identifying tissue origin in cases currently classified as CUP. The test could be a valuable addition or alternative to current diagnostic methods for classifying uncertain primary cancers. Further studies evaluating the impact of gene expression-based test results on therapy choice and treatment outcome for CUP patients are warranted.

## Abbreviations

(CUP): cancer of unknown primary; (TOO): tissue of origin; (IHC): immunohistochemistry; (SS): Similarity Score.

## Competing interests

FAM and FM were recipients of sponsored research agreements from Pathwork Diagnostics for the performance of this study. FAM has received honoraria from Pathwork Diagnostics for speaking engagements related to the Pathwork Tissue of Origin Test. WDH is an employee and stock holder of Pathwork Diagnostics. Pathwork Diagnostics financed writing/editorial assistance and article-processing charges for this manuscript.

## Authors' contributions

FAM participated in the conception, study design, sample identification, results interpretation and correlation with clinical information and manuscript writing. FM participated in the study design, sample identification, results interpretation and correlation with clinical information and manuscript writing. MALW performed all experiments for samples from University of Pittsburgh and performed acquisition and primary analysis of data. WDH participated in the conception, study design, and manuscript preparation. All authors read and approved the final manuscript.
